# Synthesis and Luminescence Properties of Water Soluble α-NaGdF_4_/β-NaYF_4_:Yb,Er Core–Shell Nanoparticles

**DOI:** 10.1186/s11671-017-2306-3

**Published:** 2017-09-25

**Authors:** Huan Chen, Peipei Zhang, Haining Cui, Weiping Qin, Dan Zhao

**Affiliations:** 1 State Key Laboratory on Integrated Optoelectronics, College of Electronic Science & Engineering, Jilin University, Changchun, 130012 China; 2 College of Physics, Jilin University, Changchun, 130012 China

**Keywords:** Upconversion, Heterogeneous-core-induced method, Hexagonal NaREF_4_, Water soluble

## Abstract

Hexagonal phase (β) sodium rare earth tetrafluorides (NaREF_4_, RE = Y, Gd, Lu, et al.) are considered the ideal matrices for lanthanide (Ln) ions doped upconversion (UC) luminescence materials, because they can provide favorable crystal lattice structures for the doped luminescent Ln ions to make intensive emissions. However, the cubic phase (α) NaREF_4_ always preferentially forms at low reaction temperature in short time as it is dynamically stable. Therefore, it is hard to obtain small sized β-NaREF_4_ via the traditional solvothermal method. In this paper, small sized β-NaYF_4_:Yb,Er nanoparticles were synthesized by a heterogeneous-core-induced method via the solvothermal reaction. The heterogeneous α-NaGdF_4_/β-NaYF_4_: Yb, Er core–shell structure was confirmed by the local elemental mapping. The formation mechanism of β-NaYF_4_:Yb,Er shell on the surface of α-NaGdF_4_ core was explained in detail. We reasoned that a hetero interface with a lower lattice symmetric structure was produced by cation exchanges between the core and shell, which caused the preferential growth of anisotropic hexagonal phase shell. The existence of this hetero interface has also been proven by observation of Gd^3+^ UC emission.

## Background

Lanthanide (Ln)-doped rare earth upconversion (UC) luminescence nanoparticles (NPs) have been intensively studied as potential fluorescent probes in biological applications for years due to their attractive luminescence properties, such as sharp emission peaks, photo stability, absence of background noise, and large tissue penetration [1–8]. As UC fluorescence probes in biological detections and imaging, these Ln^3+^-doped UCNPs should emit strong UC luminescence under infrared excitation in order to achieve high sensitivity and resolution. In the most of cases, the UCNPs are also required to have both small size (sub-50 nm) and hydrophilic surface in order to meet the post biological functionalization [9–14]. Among numerous UC materials, Ln^3+^ activator-doped rare earth fluorides, especially hexagonal phase NaREF_4_ (RE = Y, Gd, Lu), are considered ideal candidates for probes because of their efficient UC luminescence. Therefore, large numbers of researches have been focused on the synthesis of water soluble, small-sized hexagonal NaREF_4_ nanoparticles with strong UC luminescence [15–19].

At present, several methods, such as solvothermal reaction [20–23], high-temperature thermal decomposition [24–27], and doping strategy [28–31], have been developed aiming to prepare hexagonal NaREF_4_ nanocrystals that can possess all above properties. The solvothermal method in which hydrophilic surfactants have commonly been employed, for example, polyvinylpyrrolidone (PVP) or polyethylenimine (PEI) as chelating agents, has been used to synthesize good-water-soluble NaREF_4_ nanocrystals with small size [21, 32]. But these nanocrystals usually were cubic phase which exhibits less efficient than hexagonal phase counterparts for UC luminescence. Although through extending reaction time, increasing the concentration of fluorine source, or changing synthesis approaches, hexagonal phase nanocrystals could also be obtained, however, the size of these nanocrystals would correspondingly increase up to 100 nm [33, 34]. The high-temperature thermal decomposition method was developed to successfully synthesize pure hexagonal NaREF_4_ nanocrystals with small size (even ultra-small size of sub-10 nm) [27, 35, 36]. But this synthesis method has usually employed the environmentally unfriendly mixed trifluoroacetates as precursors. Moreover, it required drastic conditions such as sufficiently high reaction temperatures (over 300 °C), rather narrow temperature window of the decomposition (less than 10 °C), waterless, oxygen-free, and inert gas protection, which was generally difficult to be reproduced. Additionally, the as-prepared nanocrystals were hydrophobic, thus requiring further surface engineering to render them hydrophilic [37, 38]. Besides the above approaches, additional doping with other Ln ions could realize the phase and size control of NaREF_4_ nanocrystals in a facile way [31]. The problem which had to be faced in doping strategy was that the additional doping ions had to be over a certain amount to trigger the cubic-to-hexagonal phase transition. However, large amount of additional Ln ions probably changed the NaREF_4_ to other NaRE’F_4_ host.

In 2014, our group reported a novel heterogeneous core/shell strategy to prepare water soluble small-sized hexagonal NaREF_4_ nanoparticles [39]. In this strategy, small cubic cores were used to induce the growth of heterogeneous hexagonal shells. Two types of core/shell nanoparticles, α-NaLuF_4_/β-NaYF_4_:Yb,Er and α-NaYF_4_/β-NaLuF_4_:Yb,Er nanoparticles, were prepared. We presumed that the heterogeneous interface between the core and shell owing to cation exchange was the key factor, which caused the shell to grow into hexagonal phase. In this work, we selected cubic NaGdF_4_ nanocrystals as cores to induce the growth of hexagonal NaYF_4_ shells according to the heterogeneous core/shell strategy mentioned above. There are several reasons for NaGdF_4_ to be chosen as core material. First, Gd^3+^ possesses a large energy gap between its ground state and excited state, making NaGdF_4_ to be a good host matrix. Pure NaGdF_4_ cannot absorb 980-nm photons directly; however, if Yb^3+^ and Er^3+^ are codoped in NaGdF_4_ matrix, nanoparticles can emit ultraviolet (UV) UC luminescence through the energy transfer processes from Er^3+^ to Gd^3+^ [40–42]. In our experiments, NaGdF_4_ cores were prefabricated and no Yb^3+^ and Er^3+^ were doped. If the UC luminescence of Gd^3+^ could be observed in core/shell nanoparticles, the formation of heterogeneous interface between core and shell would be confirmed. Second, the ions radii of Gd^3+^ (0.938 Å) is larger than Y^3+^ (0.9 Å) [31]. It is easy for Y^3+^ to substitute Gd^3+^, forming a heterogeneous interface with low lattice symmetry, in our reasoning, thus triggering the growth of β-NaYF_4_ shell. Compared using NaLuF_4_ as core material, it might need shorter reaction time to obtain β-NaYF_4_ shell by using NaGdF_4_ cores. In our experiments, the heterogeneous α-NaGdF_4_/β-NaYF_4_:Yb,Er core/shell structure was certified by various characterizations.

## Methods

### Materials

The rare earth chloride, including gadolinium chloride (GdCl_3_), yttrium chloride (YCl_3_), ytterbium chloride (YbCl_3_), and erbium chloride (ErCl_3_), was obtained from Sandong Yutai Rare Earth Technology Co., Ltd. China (all with purity > 99.9%). Sodium chloride (NaCl, AR), potassium fluoride (KF, AR), and ethylene glycol (EG, AR) were bought from Shanghai Shabo Chemical Technology Co., Ltd. China. Polyvinylpyrrolidone K-30 (PVP, 58,000 g/mol) was obtained from Aldrich. All chemicals were used as received and without further purification.

### Synthesis of NaGdF_4_ Core Nanocrystals

Metal chloride (RECl_3_, NaCl) stock solutions were prepared by dissolving the corresponding metal chloride in EG. Polyvinylpyrrolidone K-30 (PVP, 0.5 g) was dissolved in EG (10 mL) to form a transparent solution. GdCl_3_ (1 mmol) and NaCl (1 mL, 1 mmol) EG solutions were subsequently added into PVP solution under strong stirring to form a solution. KF (6 mmol) was also dissolved in EG and added dropwise into the above solution. The mixture was stirred for 1 h, transferred to a polytetrafluoroethylene autoclave, and then heated at 180 °C for 0.5 h. After cooling to room temperature, the products were obtained by centrifugation and washed with deionized water and ethanol several times in order to remove the residual Gd^3+^ in the solution. The resulting products were dispersed in 10 mL EG as core for further synthesis.

### Synthesis of Heterogeneous NaGdF_4_/ NaYF_4_:Yb,Er Core/Shell Nanoparticles

PVP (0.5 g) was dissolved in the α-NaGdF_4_ core (0.5 mmol) solution. Then, YCl_3_ (0.78 mmol), YbCl_3_ (0.2 mmol), ErCl_3_ (0.02 mmol), and NaCl (5 mmol) EG solutions were added respectively under stirring. KF (6 mmol) was dissolved in EG (7 mL) and was subsequently added dropwise into above mixture. After stirring for 1 h, the solution was then transferred into a polytetrafluoroethylene autoclave and reacted at 180 °C for 2 (6, 12, 24) hours. The final product was obtained by centrifugation and washed with ethanol for several times. Half of the final product was dried in vacuum oven at 80 °C for XRD, TEM detection. The other half counterpart was redispersed in water to get the clear solution.

### Characterization

X-ray powder diffraction (XRD) analysis was carried out with a powder diffractometer (Model Rigaku RU-200b), using Ni-filtered Cu Kα radiation (*λ* = 1.5406 Å) with 200 mA current and 50 kV voltage across the tube to generate powerful X-ray. The XRD measurement was performed at a scan rate of 18°min^−1^ and step size of 0.02°. Scanning electron microscopy (SEM) measurements were carried out by a JEOL JEM-7500F Field Emission SEM. Transmission electron microscopy (TEM) and high-resolution transmission electron microscopy (HRTEM) were recorded on an FEI Tenai F-20 microscopy with a field emission gun operating at 200 kV. Images were acquired digitally by a CCD camera. The local elemental mapping and elemental compositions were determined by energy-dispersive X-ray spectrometry (EDX) under the HR-TEM mode. The UC emission spectra were recorded by a fluorescence spectrometer (Hitachi F-4500) equipped with a 980-nm diode laser. The temporal properties were studied by using a 953.6-nm Raman shifter laser and an oscillograph. The UC luminescent photos of the aqueous solution were taken by a Nikon digital camera (D300s). The digital photographs were taken using identical camera settings and same pumping power. All the measurements were performed at room temperature.

## Results and Discussion

**Fig. 1:**
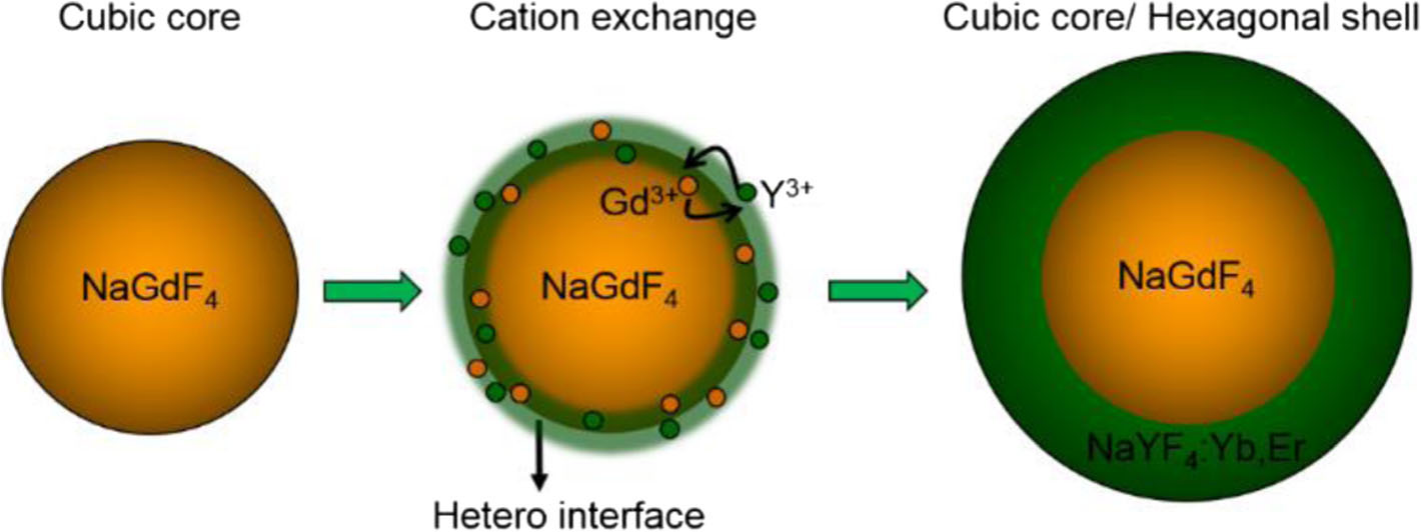
Schematic diagram of growth process of heterogeneous core/shell nanocrystals

In this work, we selected cubic NaGdF_4_ nanocrystals as cores to induce the growth of hexagonal NaYF_4_ shells using the heterogeneous core/shell strategy. The heterogeneous-core-mediated method provided a convenient route for facile synthesis of water soluble small-sized hexagonal NaYF_4_ nanocrystals. Commonly, the NaREF_4_ crystal exists two polymorphs, cubic and hexagonal phase. The structure of cubic phase NaREF_4_ is isomorphic with CaF_2_ (fluorite structure), which contains one type of high-symmetry cation site. In contrast, the crystal structure of hexagonal phase NaREF_4_ consists of two types of relatively low-symmetry cation sites. In the growth process of NaREF_4_ crystals, the cubic phase is dynamically stable and preferentially formed. In addition, with a decrease in the particle size, the crystal lattice tends to transform into a structure with higher symmetry. The above nature implies small-sized NaREF_4_ nanocrystals are cubic phase in general terms and need extra driven force to surpass the energy barrier to realize the transformation from cubic to hexagonal phase. We adopted the cubic heterogeneous core to induce the growth of hexagonal phase shell. The core and shell was different NaREF_4_. The ionic radii of two kinds of RE ions are closed but different, which means the interfacial energy is small and the heterogeneous core/shell structure can easily be built, but the mismatch intrinsically exists if the heterogeneous interface forms. Due to cation exchange, the heterogeneous interface should be formed when the shell epitaxially grows onto the core [43, 44]. The lattice symmetry of the heterogeneous interface is low because of the mismatch between the core and shell materials, thus triggering the shell subsequently grown with a low symmetric structure, i.e., hexagonal phase. According to the *Frank–van der Merwe* mode for core–shell heterostructure, once the shell material nucleate heterogeneously onto a pre-existing condensed core, the energy barrier which has to be surpassed is lower than the activation energy which is required to induce the corresponding homogenous nucleation of separate crystal nuclei. It indicates that building a core–shell heterostructure can efficiently decrease the energy barrier, which benefits to the growth of thermodynamically stable β-NaYF_4_ shell with lower free energy rather than the formation of homogenous α-NaYF_4_ embryos with higher activation energy. Considering the above factors, we adopt the core/shell heterostructure strategy to control the dynamical process of crystal growth as well as decrease the lattice symmetry of crystal interface to synthesize water soluble β-NaYF_4_ nanocrystals with small size at low temperature. The schematic diagram of growth process of α-NaGdF_4_/β-NaYF_4_:Yb,Er nanocrystals is shown in Fig. 1. In the first step, we synthesized small-sized NaGdF_4_ nanocrystals by solvothermal reaction at low temperature of 180 °C. Then, we introduced the cubic NaGdF_4_ nanocrystals into a NaYF_4_ precursor solution containing Yb^3+^ and Er^3+^. In the second solvothermal reaction process, cation exchange firstly occurred. Y^3+^ entered into the NaGdF_4_ matrix and exchange with Gd^3+^ that located in the crystals surface, forming a hetero interface containing both Gd^3+^ and Y^3+^ on the surface of the core crystals. Because of the ion radii of Gd^3+^ and Y^3+^ are different, it would cause the lattice distortion of the hetero interface, forming a low symmetric structure. Additionally, according to above presentation, the energy barrier of the shell crystals formation could be efficiently decreased by building a heterostructure. Therefore, it would cause the preferential growth of anisotropic hexagonal phase NaYF_4_ shell with relatively low symmetry following the hetero interface layer by surpassing a lower energy barrier, forming the heterogeneous α-NaGdF_4_/β-NaYF_4_:Yb^3+^,Er^3+^ core/shell nanoparticles.

**Fig. 2:**
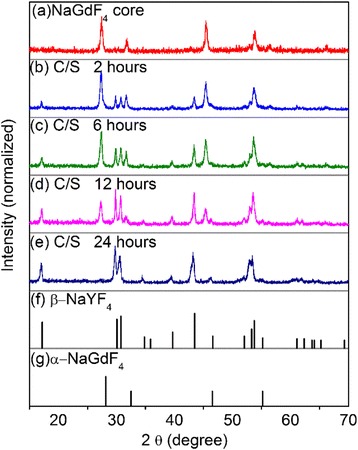
XRD patterns of (**a**) the NaGdF_4_ core nanocrystals, and the NaGdF_4_/ NaYF_4_:Yb,Er core/shell (named C/S in the figure) nanocrystals with different reaction time, (**b**) 2 h, (**c**) 6 h, (**d**) 12 h, and (**e**) 24 h. (**f**) Standard data of β-NaYF_4_ (JCPDS 16-334). (**g**) Standard data of α-NaGdF_4_ (JCPDS 27-697)

To carry out our idea, we synthesized NaGdF_4_ core nanocrystals, acting as induced reagents, for the subsequent growth of β-NaYF_4_ shells. In order to study the growth process of the hexagonal shell, we prepared a set of heterogeneous core/shell crystals by varying the second-step reaction time (shell growth time) from 2 to 24 h. All the reactions in our experiment were based on a solvothermal method using polyvinylpyrrolidone (PVP) as the chelating agent. Both the core and the final core/shell products were examined by XRD to determine the crystal structures. Figure 2 showed the XRD patterns of NaGdF_4_ core nanocrystals (Fig. 2a) and a set of heterogeneous NaGdF_4_/NaYF_4_:Yb,Er core/shell nanocrystals with reaction time of 2, 6, 12, and 24 h (Fig. 2b–e). It can be seen that the core crystals were indexed as pure cubic phase NaGdF_4_ crystals (Fig. 2g; α-NaGdF_4_: JCPDS file number 27-697). After subsequent solvothermal treatment with NaYF_4_ precursors for 2 h, in the XRD pattern (Fig. 2b), there were extra diffraction peaks appeared besides the diffraction peaks of α-NaGdF_4_. All these new peaks were well consistent with the standard β-NaYF_4_ crystals (Fig. 2f; β-NaYF_4_: JCPDS file number 16-334), instead of β–NaGdF_4_. With the reaction time increasing to 6, 12, and 24 h, the intensities of hexagonal NaYF_4_ diffraction peaks gradually increased. It indicated that the fraction of the hexagonal phase counterparts in the shells were gradually increased as the reaction proceeding. Note that, for 24-h-reaction samples, the hexagonal NaYF_4_ diffraction peaks were so intensive that the cubic NaGdF_4_ peaks were almost not observed (Fig. 2e). The reason may be that the hexagonal NaYF_4_ shell grew so thick that the strong hexagonal signals pressed down the cubic phase signals, leading to the seemed-absence of α-NaGdF_4_ diffraction peaks. In a word, the XRD results indicated the formation of hexagonal NaYF_4_ crystals which become more and more dominant with the reaction time increasing.

**Fig. 3:**
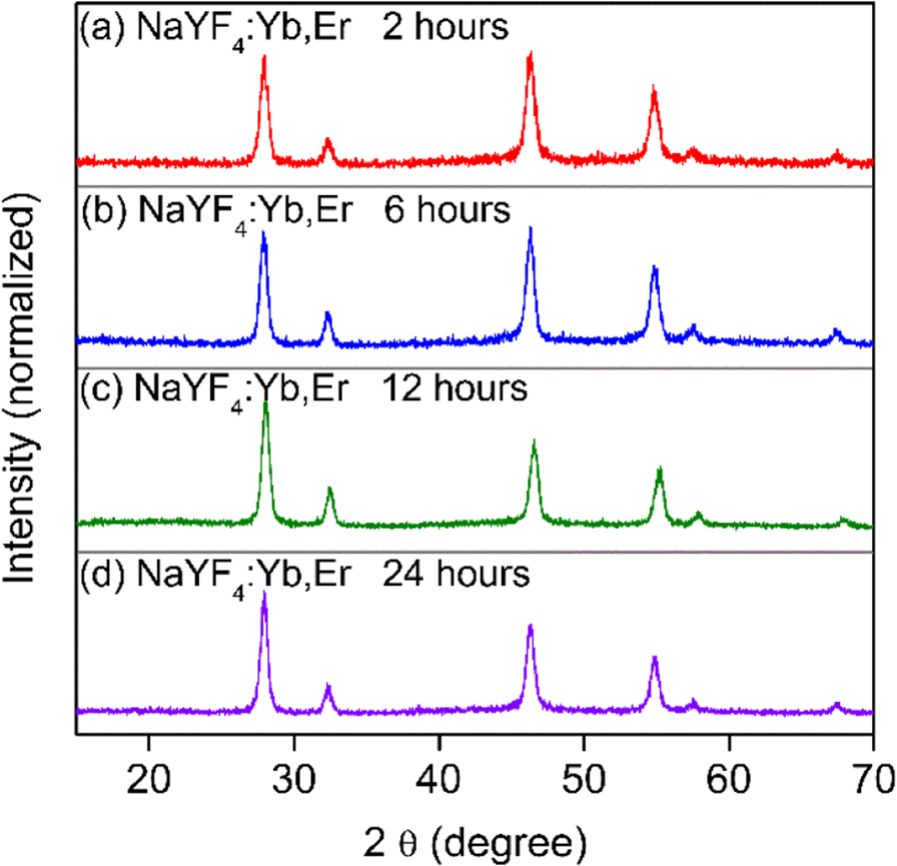
XRD patterns of NaYF_4_: Yb, Er crystals which were synthesized without α–NaGdF_4_ core existing with different reaction times. (**a**) 2 h, (**b**) 6 h, (**c**) 12 h, and (**d**) 24 h

We also performed another set of contrast experiments that we synthesized the NaYF_4_ crystals without α-NaGdF_4_ core existing, keeping all other reaction conditions the same as the preparation of NaGdF_4_/NaYF_4_ nanocrystals. The crystal structures were examined by XRD. As shown in Fig. 3a, the diffraction peaks of the 2-h-reaction NaYF_4_:Yb,Er crystals were indexed to cubic NaYF_4_ crystals (JCPDS file number 77-2042). With reaction time increasing to 6 and 12 h, even to 24 h (Fig. 3b–d), the as-prepared NaYF_4_ crystals still keep the pure cubic phase, instead of appearance of any hexagonal phase NaYF_4_ signals. These results indicated that, in this low-temperature solvothermal reaction system, the α–NaGdF_4_ core crystals were the requirement for growth of hexagonal phase NaYF_4_ crystals.

**Fig. 4:**
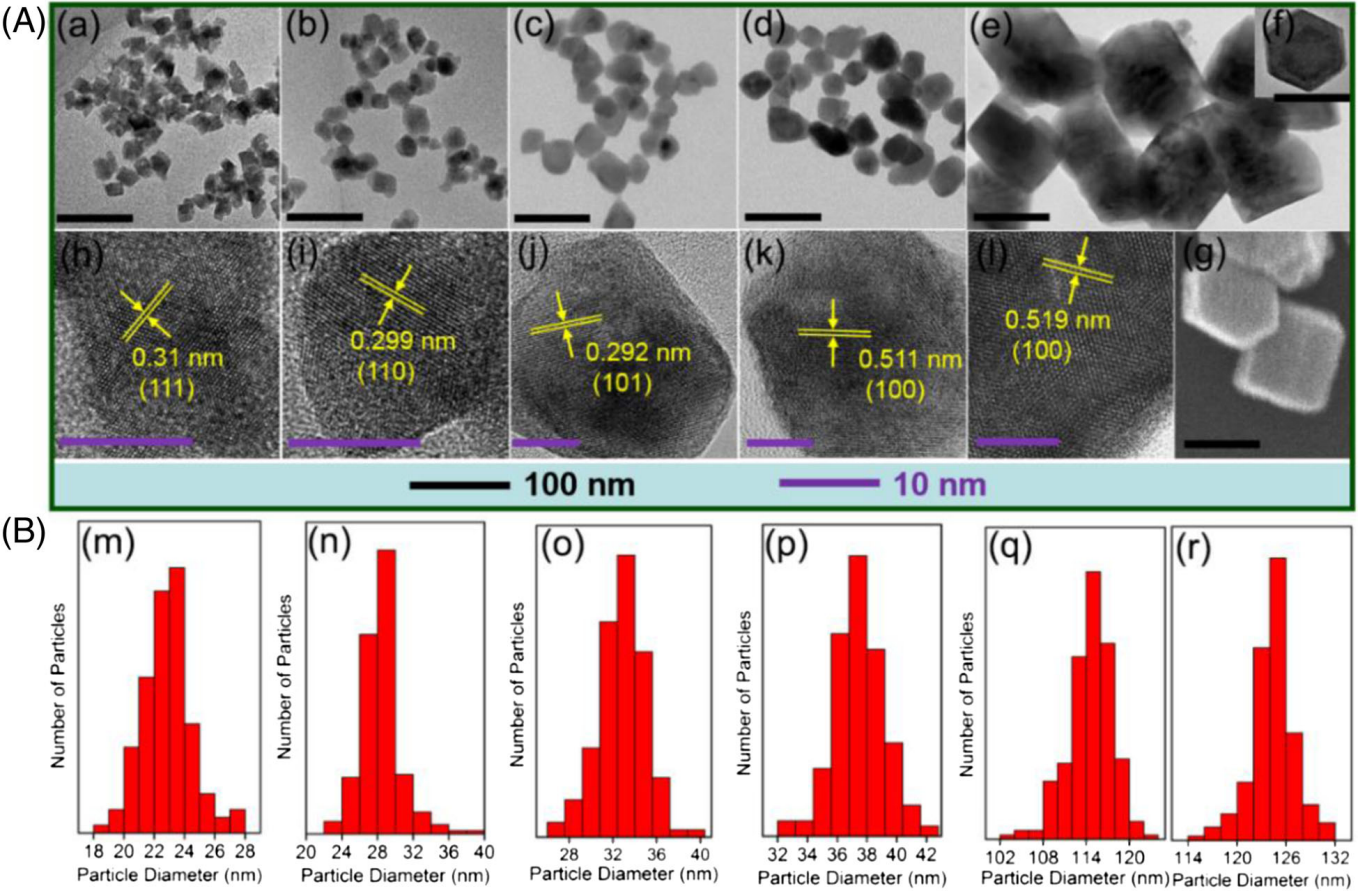
**A** TEM and HRTEM images of the as-prepared cubic core crystals and heterogeneous core/shell crystals. TEM images of (a) cubic NaGdF_4_ core nanocrystals, (b)–(e) heterogeneous NaGdF_4_/ NaYF_4_ core/shell crystals with the shell growth time of 2, 6, 12, and 24 h, respectively, (f) a single particle of the 24-h-reaction NaGdF_4_/ NaYF_4_ core/shell crystals which stand on the TEM grids on their bottom faces. (g) SEM image of the 24-h-reaction NaGdF_4_/NaYF_4_ core/shell crystals, which exhibit a hexagonal nanoprism look. HRTEM images of (h) cubic NaGdF_4_ core nanocrystals. (i)–(l) heterogeneous NaGdF_4_/NaYF_4_ core/shell crystals with the shell growth time of 2, 6, 12, and 24 h, respectively. **B** Histograms of size distribution of (m) cubic NaGdF_4_ core nanocrystals, (n)–(p) heterogeneous NaGdF_4_/NaYF_4_ core/shell crystals with the shell growth time of 2, 6, and 12 h, respectively, (q) and (r) diameter of the hexagonal cross section and the nanoprism height of the 24-h-reaction NaGdF_4_/ NaYF_4_ core/shell crystals

The morphologies and size distributions of the core and core/shell samples are shown in Fig. 4. We performed TEM to characterize the morphologies of the cubic NaGdF_4_ core nanocrystals (Fig. 4A (a)) and heterogeneous NaGdF_4_/NaYF_4_ core/shell crystals with the shell growth time of 2, 6, 12, and 24 h, respectively (Fig. 4A (b–f)). The corresponding size distributions were shown in Fig. 4B. From the TEM image of the cubic NaGdF_4_ core nanocrystals (Fig. 4A (a)), we can see that the particles were nearly uniform square shape, with average size about 23 nm (corner to corner), which was shown in the histogram of size distribution (Fig. 4B (m)). The HRTEM image displayed the clear lattice fringes pattern of the core crystals (Fig. 4A (h)), and the interplanar spacing of 0.31 nm was indexed to (111) plane of cubic NaGdF_4_. After subsequent growth of NaYF_4_ shell for 2 h, the particle shapes varied that the nanosquare edges became somewhat rounded and the particle size increased to 28 nm on average (Fig. 4B (n)). The lattice fringes were shown in HRTEM image, in which the measured interplanar spacing of 0.299 nm was indexed to (110) plane of hexagonal NaYF_4_ crystal. Combining the TEM, HRTEM, and XRD results, for 2-h-reaction samples, the growth of NaYF_4_ shell with thickness of 2.5 nm and the appearance of hexagonal NaYF_4_ lattice fringes and diffraction peaks could preliminarily confirmed the formation of hexagonal NaYF_4_ shells on the cubic NaGdF_4_ cores. Further prolonging the reaction time to 6 and 12 h, the particle size continued increasing to 33 and 38 nm, respectively, and the particle shape varied to nanopolyhedron, enclosed by the (101) and (100) planes, respectively (Fig. 4A (j, k)). Note that the increase of shell thickness were in agreement with the data in XRD that the hexagonal NaYF_4_ diffraction peaks became more and more intensive, which further implied that the NaYF_4_ crystals existed in the form of hexagonal shell. Then, extending the reaction time to 24 h, we found that, from 12 to 24 h, the crystals grew rapidly to the hexagonal prism shape, which is shown in the TEM images (Fig. 4A (e, f)) and SEM image (Fig. 4A (g)). We can see the hexagonal nanoprism stood on the TEM grids either on their bottom faces or on their side faces. The mean size was about 115 nm × 125 nm (diameter from corner to corner × height, shown in Fig. 4B (q, r)) and the hexagonal NaYF_4_ lattice fringes were also displayed clearly in HRTEM image (Fig. 4A (l)) that the interplanar spacing of 0.519 nm was indexed to (100) plane. For the 24-h-reaction heterogeneous NaGdF_4_/NaYF_4_:Yb,Er core/shell samples, the shell grew so thick (~ 50 nm) that, consistently, in above XRD pattern (Fig. 2e), the hexagonal NaYF_4_ peaks were dominant and the cubic NaGdF_4_ peaks almost cannot be observed.

**Fig. 5:**
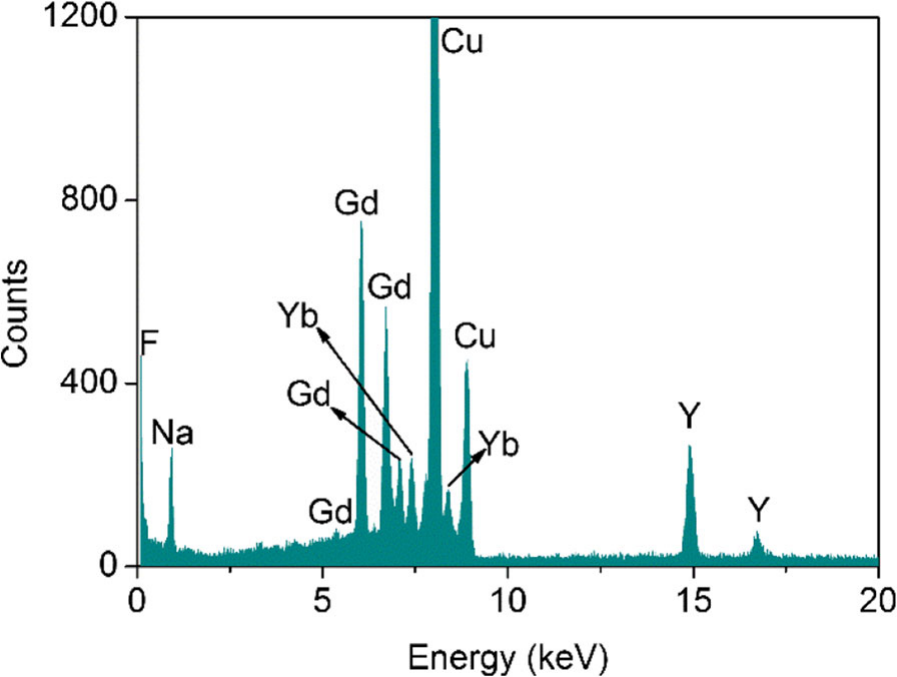
EDX analysis of elemental composition of heterogeneous NaGdF_4_/ NaYF_4_ core/shell crystals

EDX analysis was performed to examine the elemental composition of the heterogeneous NaGdF_4_/NaYF_4_ core/shell crystals. Figure 5 showed the EDX pattern of 6-h-reaction samples. The major elements of Gd, Y, Yb, Na, and F were observed in the pattern, while Er element was not observed because of the ultra-little dopant concentration of 2%.

**Fig. 6:**
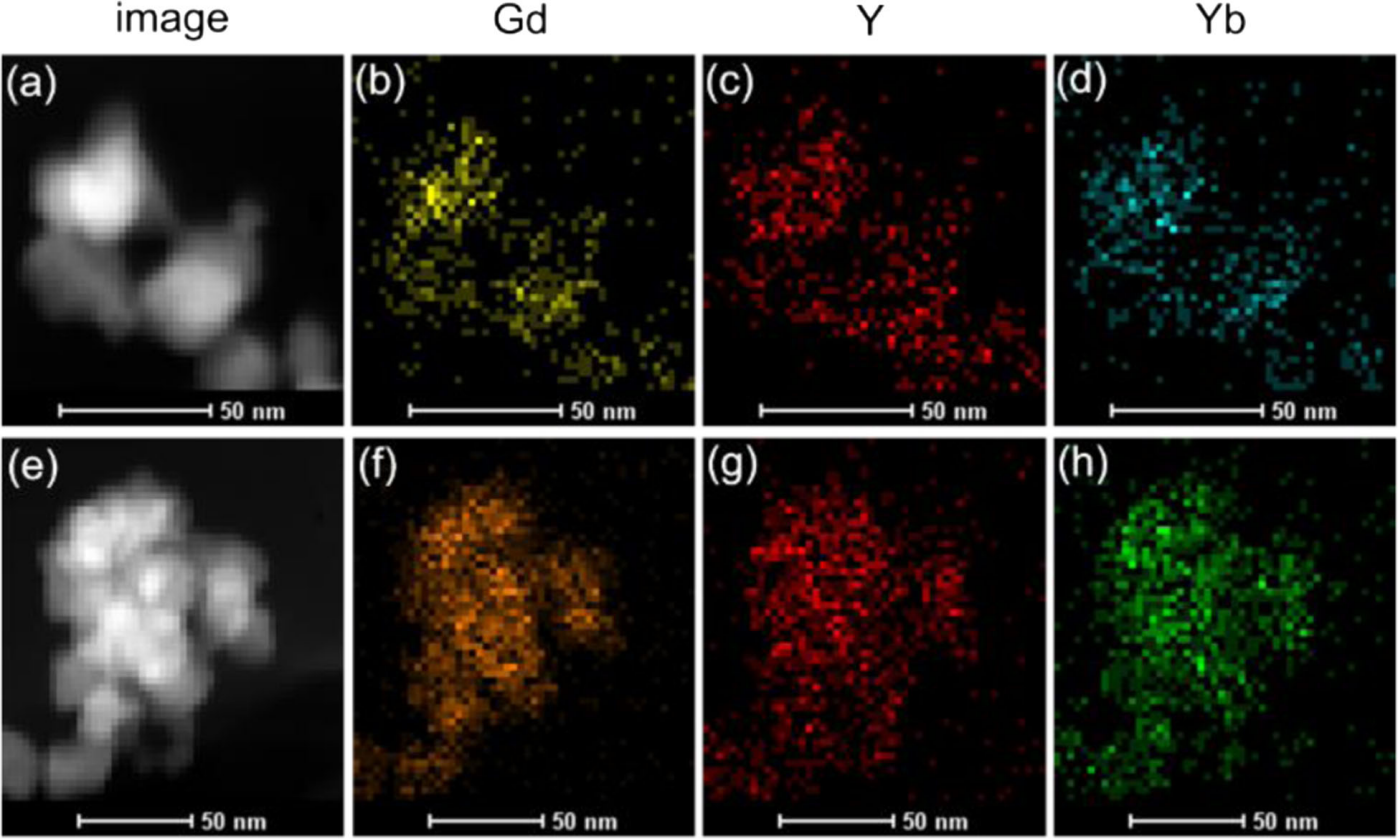
Images of local elemental mapping by EDX line scans for **a**–**d** the 2-h-reaction NaGdF_4_/NaYF_4_:Yb,Er crystals and **e**–**h** the 6-h-reaction NaGdF_4_/NaYF_4_:Yb,Er crystals. **a**, **e** image of EDX line scans areas. **b**, **f** Gd in core. **c**, **g** Y in shell. **d**, **h** Yb in shell

To further confirm the heterogeneous core/shell geometry of the as-prepared α-NaGdF_4_/β-NaYF_4_ crystals, the local elemental mapping by EDX line scans were carried out to address the composition across of the nanocrystals. The images of EDX elemental mapping are given in Fig. 6, in which (a)–(d) were the image of selected scan area, Gd, Y, and Yb, respectively, of the 2-h-reaction samples, and (e)–(h) were the image of selected scan area, Gd, Y, and Yb, respectively, of the 6-h-reaction samples. For the two crystal samples, we observed that all the three examined elements of Gd, Y, and Yb are homogenously dispersed overlap the nanocrystals, and the labeled areas of Gd element were a little smaller than that of Y and Yb elements. The fact suggested that Y and Yb elements uniformly covered across the whole outer layer of the nanocrystals, while Gd element was localized in the center. These elemental mapping results were strong proofs of the heterogeneous core/shell structure formation of the α-NaGdF_4_/β-NaYF_4_:Yb,Er crystals.

**Fig. 7:**
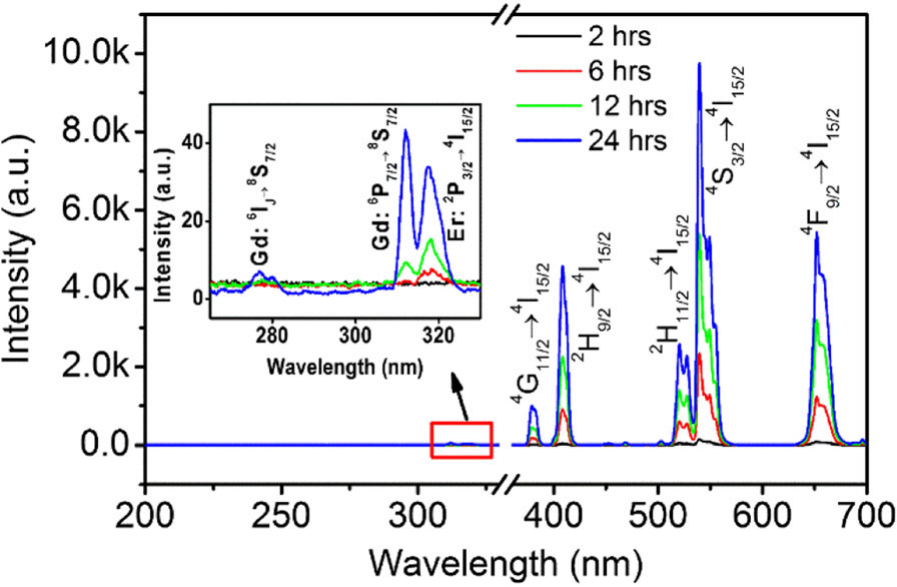
UC luminescence spectra of the as-prepared heterogeneous core/shell α-NaGdF_4_/β-NaYF_4_:Yb,Er crystals with varied reaction time (black: 2 h, red: 6 h, green: 12 h, blue: 24 h) excited by 980-nm infrared light. (Inset: Local magnified patterns of UC spectra in the region of 265–330 nm, in which the characteristic peaks of Gd^3+^ were observed)

Next, the optical properties of the heterogeneous α-NaGdF_4_/ β-NaYF_4_: Yb,Er core/shell crystals were characterized and discussed. Co-doping Yb^3+^ (20 mol%) and Er^3+^ (2 mol%) in the hexagonal NaYF_4_ shell could realize efficient UC luminescence. Under 980-nm excitation, Yb^3+^ ions can absorb infrared photons and transfer the energy to Er^3+^ ions successively. The excited Er^3+^ ions then emit characteristic UC luminescence in the region of 300–700 nm. Figure 7 showed the UC luminescence spectra of the *x*-hours-reaction (*x* =changed the NaREF4 to other 2, 6, 12, 24) α-NaGdF_4_/β-NaYF_4_: Yb, Er crystals under 980-nm excitation, with the same pumping power of 160 mW (pumping power density of 16 W cm^−2^). In each spectrum of the as-prepared samples, there were all the characteristic UC peaks of Er^3+^ ions. We observed that with the shell growth time increasing, the overall emission intensities increased, while the intensity ratios of every emission peaks almost unchanged. The green emission peaks in the range of 515–560 nm from the ^2^H_11/2_, ^4^S_3/2_ → ^4^I_15/2_ transitions were relatively strong, while the blue emission peaks from the ^2^H_9/2_ → ^4^I_15/2_ transitions centered at 409 nm and the red emission peaks between 640 and 680 nm, corresponding to the ^4^F_9/2_ → ^4^I_15/2_ transitions, were relatively weak. Interestingly, several so-weak emission peaks centered at 317, 312, and 277 nm were observed, which originated from Er^3+^: ^4^P_3/2_ → ^4^I_15/2_, Gd^3+^: ^6^P_7/2_ → ^8^ s_7/2_, and Gd^3+^ [6]:I_J_ → ^8^ s_7/2_ (inset of Fig. 7). It is known that Gd^3+^ ions cannot absorb 980-nm photons directly; therefore, there must exist energy transfer processes from Er^3+^ to Gd^3+^ at high energy excited states, which induced the UC emissions of Gd^3+^ ions. In our designed core/shell α-NaGdF_4_/β-NaYF_4_: Yb, Er crystals, Gd^3+^ ions were located in the core and Yb^3+^, Er^3+^ ions existed in the shell; thus reasonably, the energy transfer processes from Er^3+^ to Gd^3+^ should occur in the hetero interface between the α-NaGdF_4_ core and β-NaYF_4_ shell. Inspiringly, this is a proof to certify the existence of the hetero interface which attributed to the hexagonal shell growth in our reasoning.

**Fig. 8:**
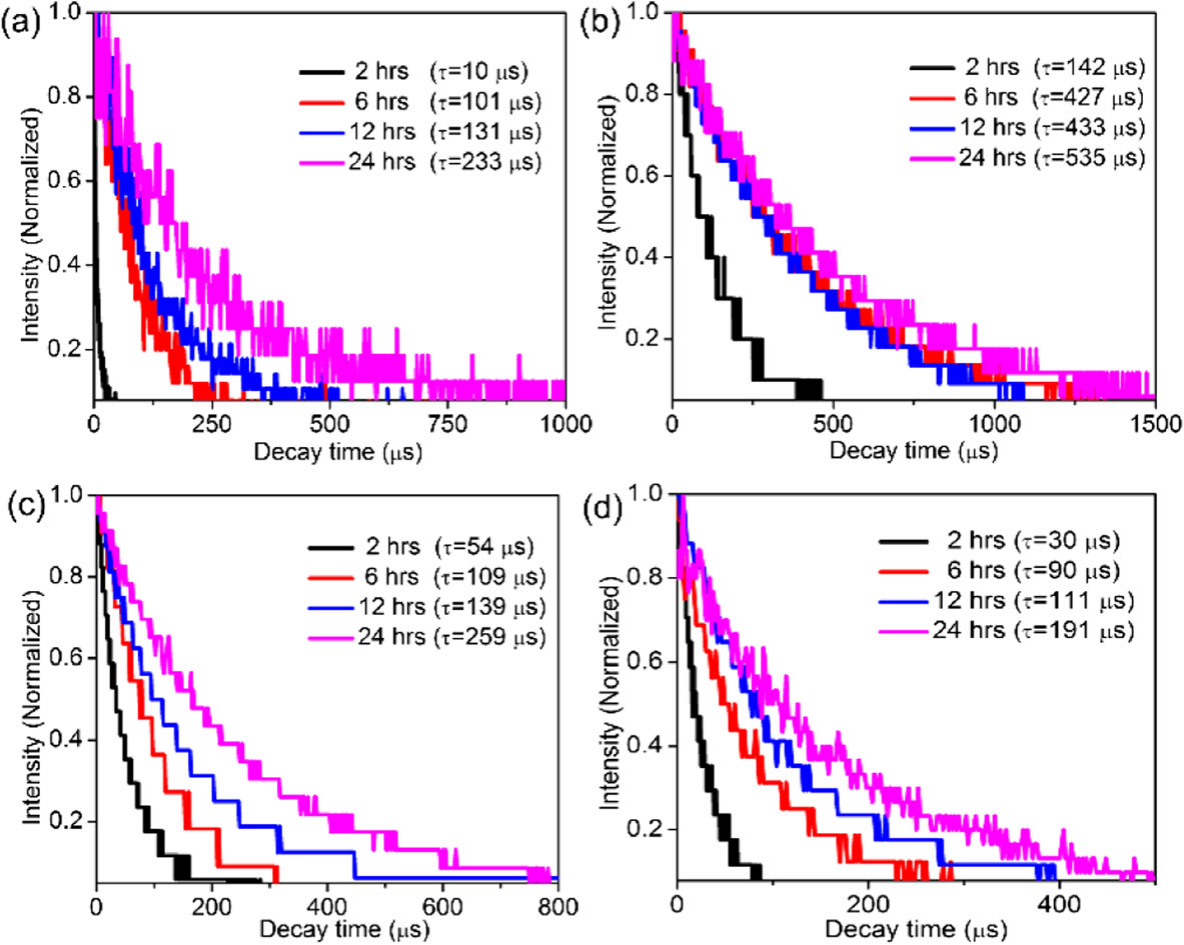
Photoluminescence decay curves of the **a** ^2^F_5/2_ level of Yb^3+^, **b** ^4^F_9/2_ level of Er^3+^, **c** ^4^F_9/2_ level of Er^3+^, and **d** ^2^H_9/2_ level of Er^3+^ in the heterogeneous core/shell α-NaGdF_4_/β-NaYF_4_:Yb, Er crystals with varied reaction time (black: 2 h, red: 6 h, blue: 12 h, pink: 24 h)

In addition, we measured the lifetimes of various levels of Yb^3+^ and Er^3+^ in *x*-hours-reaction (*x* = 2, 6, 12, 24) α-NaGdF_4_/β-NaYF_4_:Yb,Er core/shell crystals, using a 953.6-nm pulsed laser with a pulse width of 10 ns and a repetition rate of 10 Hz as the excitation source. Figure 8 showed the photoluminescence decay curves of the ^2^F_5/2_ level of Yb^3+^ (Fig. 8a) and ^4^F_9/2_, ^4^F_9/2_, ^2^H_9/2_ levels of Er^3+^ (Fig. 8b–d) in the *x*-hours-reaction (*x* = 2, 6, 12, 24) α-NaGdF_4_/β-NaYF_4_: Yb, Er core/shell crystals. All the decay curves could be well fitted by a single exponential function *I*_*(t)*_ = *I*_0_ exp.(−t/τ), where *I*_0_ is the initial emission intensity at *t* = 0 and *τ* is the lifetime of the monitored level. The measured lifetime data of all monitored levels in each detected samples were listed in Fig. 8. Obviously, for all monitored levels, the lifetime gradually increased with the shell growth time increasing from 2, to 6, to 12, to 24 h. For example, the measured lifetime of ^4^F_9/2_ level of Er^3+^ is 54, 109, 139, and 259 μs for 2, 6, 12, and 24-h-reaction α-NaGdF_4_/β-NaYF_4_: Yb, Er core/shell crystals, respectively (Fig. 8c). These results were consistent with the UC luminescence spectra data that the overall emission intensities increased with the shell growth time increasing. The reason was that the β-NaYF_4_: Yb, Er shell counterpart which provided UC luminescence was increased with the reaction being prolonged.

## Conclusions

In summary, a heterogeneous core/shell structure was constructed, in which cubic NaGdF_4_ nanocrystals, serving as cores, induced the growth of the heterogeneous hexagonal NaYF_4_ shells co-doped with Yb^3+^ and Er^3+^ ions. We prepared a set of heterogeneous α-NaGdF_4_/β-NaYF_4_:Yb,Er core/shell particles by varying the shell growth time from 2 to 24 h. The characterizations of XRD, TEM, HRTEM, EDX, and local elemental mapping results were performed. With the growth time increasing from 2 h, to 6 h, to 12 h, and finally to 24 h, the diffraction peaks of hexagonal NaYF_4_ in the XRD patterns became more and more dominant, and the crystals shapes changed from nanosquare, to nanopolyhedron, and finally to hexagonal prism shape, and the particle size increased from 28 nm, to 33 nm, to 38 nm, and finally to 115 nm × 125 nm, respectively. Moreover, in the local elemental mapping images, Gd element was localized in the center, and Y and Yb elements uniformly covered across the whole outer layer of the nanocrystals. All the results confirmed the successful formation of the unique heterogeneous α-NaGdF_4_/β-NaYF_4_:Yb,Er core/shell particles. Moreover, the existence of hetero interface between cubic cores and hexagonal shells was confirmed by the observation of Gd^3+^ UC emission. In our reasoning, we demonstrated that the hetero interface, which were produced by cation exchanges that caused a lower lattice symmetric structure, attributed to the heterogeneous hexagonal shell growth.
